# Time Course and Predictors of Persistent Postoperative Dysphagia in Patients with Congenital Heart Disease Following Cardiac Surgery

**DOI:** 10.1007/s00246-025-03892-8

**Published:** 2025-05-29

**Authors:** Susan Willette, Sarah Hahn, Amy Lay, James Schroeder, Inbal Hazkani, Taher Valika, Saied Ghadersohi

**Affiliations:** 1 Department of Speech Pathology, Ann and Robert H. Lurie Children’s Hospital of Chicago, Chicago, IL USA; 2 Department of Pediatric Cardiology, Ann and Robert H. Lurie Children’s Hospital of Chicago, Chicago, IL USA; 3 Division of Pediatric Otolaryngology – Head and Neck Surgery, Ann and Robert H. Lurie Children’s Hospital of Chicago, 225 East Chicago Ave, Box #25, Chicago, IL 60611 USA; 4 Department of Otolaryngology Head and Neck Surgery, Feinberg School of Medicine, Northwestern University, Chicago, IL USA

**Keywords:** Congenital heart disease, Cardiac surgery, Dysphagia, Aspiration, Vocal fold motion impairment, Pediatric feeding

## Abstract

Describe the long-term presence, predictors, and time course of postoperative dysphagia in selected congenital heart disease (CHD) patients following cardiac surgery. Retrospective study of selected CHD patients who underwent cardiac surgery that is at high risk for dysphagia and vocal fold mobility impairment (VFMI) and underwent speech pathology assessment from 2019 to 2024. Demographics, clinical history, VFMI, dysphagia severity and feeding modality were assessed. There were 322 mostly infant patients; median age was 0.64 (IQR0.19–6.9) months. Most patients were male (177,55%) and 119 (37%) had single ventricle (SV) disease. A full PO diet was maintained in 105 (32.6%) patients throughout follow-up. One hundred and ten (34.2%) patients improved from tube feeding (TF) at initial discharge to a full PO diet in a median 10.4 months (IQR4.4–23.2), whereas 107 (33.2%) remained on TFs with severe dysphagia at the end of follow-up (median 7.2, IQR1.5–17.3 months). VFMI was present in 83 patients postoperatively. VFMI resolved in 35 (38.8%) patients in a median 4.8 months (IQR2.4–8.2). VFMI and dysphagia recovery were not associated. However, the time to VFMI recovery and time to dysphagia resolution were correlated (*r* = 0.77, *p* = 0.0001). Patients with a genetic syndrome, Blalock–Thomas-Taussig shunt and/or ventricular assist device use were more likely to require persistent TF. Patients who had coarctation/aortic arch repair or SV disease were less likely to require TF at the end of follow-up. Dysphagia necessitating tube feeding persists for several months after VFMI resolves. This study will help set prognostic expectations for caregivers.

## Introduction

Dysphagia and feeding difficulties can be present after surgical repair of congenital heart disease (CHD) in 18–83% of children [1–3]. The patient’s gestational age, age at the time of surgery, their underlying neurological status, the presence of a genetic syndrome, the surgical procedure that was performed, and the patient’s respiratory stability including the length of intubation all impact the risk for and severity of dysphagia [4–6]. Hoffmeister et al. demonstrated extubation-related dysphagia in 29% of patients especially in those less than 25 months old, with the odds of dysphagia increasing by 1.7% for every additional hour of intubation required [7]. Additionally, postoperative vocal fold mobility impairment (VFMI), has been commonly reported in this patient population with an incidence that ranges from 1.7 to 67% [8, 9]. Several studies also describe a connection between VFMI and aspiration in this patient population [10–12]. Many of these children have severe dysphagia requiring alternative feeding methods including nasogastric and surgically placed gastrostomy tubes. Parental stress and the already significant financial burden for these patients can be exacerbated by feeding difficulties which can include feeding tube management, hospital readmissions related to weight loss, and feeding intolerance [13–15]. While some studies describe the time course for the resolution of VFMI, there are few studies that describe the time required for the resolution of dysphagia and discontinuation of tube feedings [14]. Richter et al., described the mean time to oral feeding for patients with VFMI to be 0.8 ± 1.4 years however did not report any recovery time for VFMI or resolution of aspiration for these patients [14].

The aim of this study is to describe the long-term presence, predictors, and time course of post-operative dysphagia in children who have undergone surgical repair of congenital heart disease (CHD) beyond the initial admission. The relationship of VFMI and dysphagia recovery and their time course are also examined.

## Materials and Methods

Approval for this study was obtained from the Institutional Review Board at The Ann & Robert H. Lurie Children’s Hospital of Chicago (IRB# 2023-6082). This is a retrospective case series of children from birth to age 21 who had surgical repair of CHD between January 1, 2019 and January 1, 2024. Using CPT codes an electronic medical record search to identify patients who had undergone the following procedures was performed: patent ductus arteriosus (PDA) ligation, Norwood procedure, Blalock–Thomas–Taussig (BTT) shunt, bidirectional Glenn, aortic arch repair, coarctation of aorta repair, aortic root replacement, vascular ring repair, arterial switch operation, truncus arteriosus repair, heart transplant and left ventricular assist device (LVAD) placement. These selected procedures were chosen as institutionally they are often associated with dysphagia and/or VFMI. In patients that had multiple surgeries, the surgery that was closest in date to the diagnosis of dysphagia or VFMI was considered the index surgery.

**Fig. 1:**
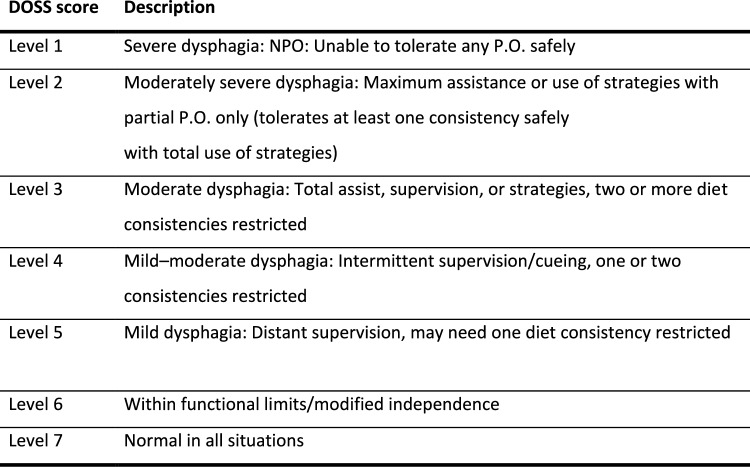
DOSS—Dysphagia outcome severity score

Dysphagia and tube feeding status of each patient was then assessed by a speech language pathologist (SLP) through chart review of the pre and postoperative clinical and/or instrumental swallow evaluations. Dysphagia for this study was operationally defined as a requirement for tube feeding at discharge. It was further qualified objectively using the Dysphagia Outcome and Severity Score (DOSS), an objective measurement based on findings on a video fluoroscopic swallow study (VFSS) or fiberoptic endoscopic evaluation of swallowing (FEES), (Fig. 1). Thickened diet recommendations (International dysphagia diet standardization initiative IDDSI) were also recorded. [16, 17]

**Fig. 2:**
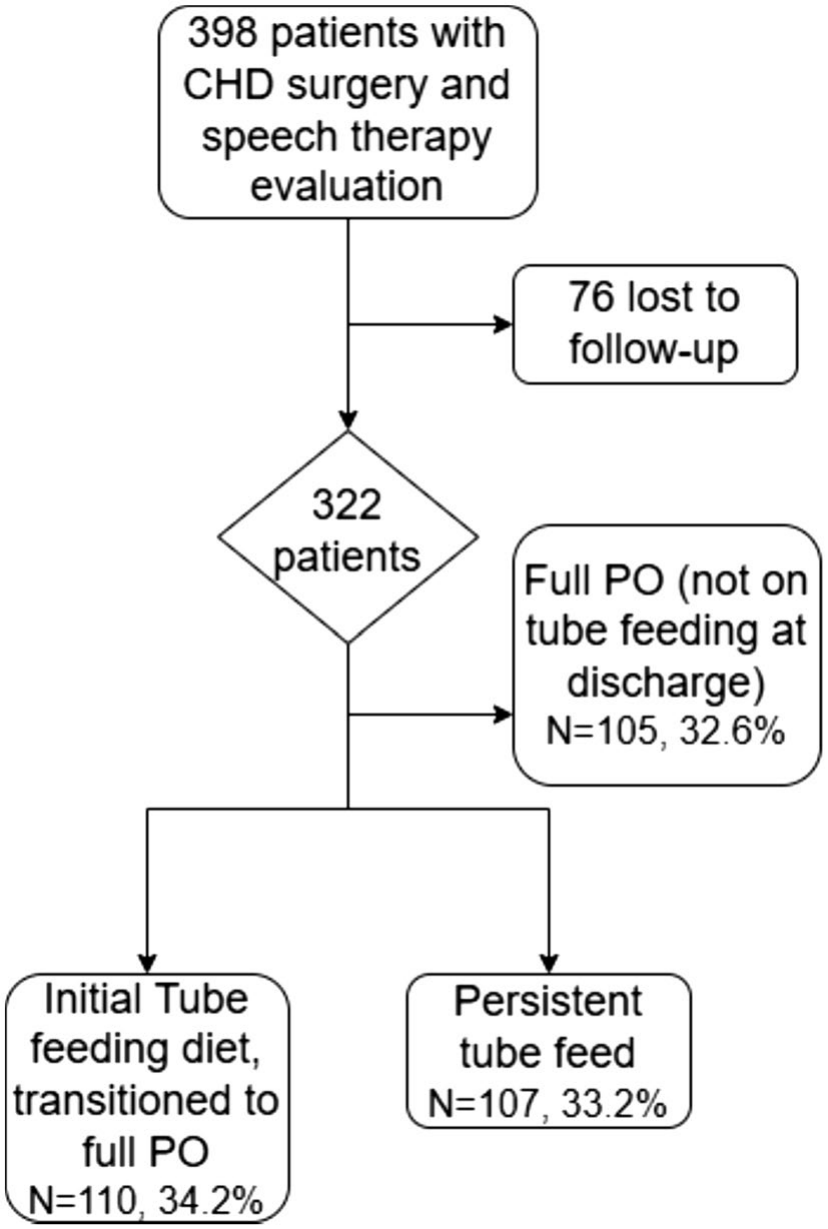
Study patient flowchart including grouping based on tube feeding vs PO diet status at initial discharge and last follow-up

The patients were grouped based on dysphagia and feeding modality at both initial discharge and end of follow-up. The patients that remained on a full PO diet at initial discharge and at the end of follow-up or in other words, did not have significant dysphagia through their recorded clinical course, were excluded. The remaining patients were grouped into two groups, those that improved from a tube feeding diet at initial discharge to a full PO diet at the end of follow-up, or those with persistent need for tube feeding at initial discharge and at the end of follow-up. (Fig. 2).

Demographic information, history of prematurity (less than 37 weeks’ gestation), and history of single ventricle cardiac disease were collected. Comorbidities associated with dysphagia including a history of extracorporeal membrane oxygenation (ECMO), history of genetic syndrome, neurologic comorbidity, airway abnormality, lower airway abnormality, tracheostomy and gastrostomy tube status were assessed. Surgical complications such as VFMI, chylothorax and diaphragm paresis/paralysis were also collected. The diagnosis of VFMI and diaphragm paresis was confirmed by fiberoptic laryngoscopy and imaging, based on postoperative clinical suspicion.

Demographic and clinical characteristics were reported as frequencies and percentages for categorical variables, means and standard deviations for continuous variables and median with interquartile range (IQR) for ordinal or parametrically distributed continuous variables. Data variables were compared with the chi-squared testing, Wilcoxon rank sum test, Students t-test where applicable. Significance was determined at *p* < 0.05. Multivariate logistic regressions were performed to assess variable effects on transition to full PO diet versus persistent need for tube feeds at the end of follow up. A stepwise procedure was used for multivariable model construction, with a *P*-value of < 0.15 (on univariable analysis) to enter the model and a *P*-value of 0.05 to remain in the model. All statistical analyses were performed using Stata 14.1 (Statacorp, College Station, TX).

## Results

### Cohort Description

We identified 398 patients who had selected types of cardiac surgeries and had swallowing information documented at discharge after surgery. Seventy-six patients (19.1%) were excluded as they were either lost to follow-up and/or did not have either a subsequent clinical or instrumental swallowing evaluation. The remaining 322 patients underwent surgery at a median age of 0.64 (IQR 0.19–6.9) months with 263 (81.2%) at less than 12 months of age and 145 (45%) at less than 1 month of age. There was a slight male predominance with 177 (55%). Single ventricle cardiac disease was present in 119 (37%) patients. Table 1 shows the baseline characteristics, specific cardiac surgery performed, comorbidities, and surgical complications. Laryngoscopy was performed after cardiac surgery in 153 (47.5%) patients based on clinical suspicion from either postoperative voice changes or aspiration on instrumental swallow study. Of the patients with laryngoscopy data, 83 (54.2%) were noted to have VFMI at initial discharge. Follow-up laryngoscopy was performed in 66 (43.1%) patients and 31 (47%) had persistent VFMI at the end of follow-up.

**Table 1:** Baseline characteristics of those with diet follow up

Number of patients, N	322
Age at surgery (months)	0.64 (IQR0.19–6.9)
Female gender	145 (45.0%)
Prematurity	31 (9.6%)
Single ventricle cardiac disease	119 (37%)
Surgical procedure	
PDA ligation	9 (2.8%)
Norwood	41 (12.7%)
PDA stent/PA banding	32 (9.9%)
BTT shunt	26 (8.1%)
Aortic root	0 (0%)
Coarctation of Aorta repair/arch repair	75 (23.3%)
Vascular ring repair	24 (7.5%)
Arterial switch operation	41 (12.7%)
Truncus arteriosus repair	6 (1.9%)
Heart transplant	37 (11.5%)
LVAD	26 (8.1%)
Other	5 (1.6%)
Comorbidities	
ECMO	14 (4.4%)
Syndromic	51 (15.8%)
Neurological	119 (37%)
Airway abnormality	34 (10.6%)
Lower airway	40 (12.4%)
Tracheostomy	35 (10.9%)
Gastrostomy tube	61 (18.9%)
Nissen	4 (1.2%)
Surgical complications	
Chylothorax	66 (20.5%)
Diaphragm paralysis	23 (7.1%)
VFMI	
Postoperative laryngoscopy performed	153 (47.5%)
VFMI after surgery	83 (54.2%)
Follow-up laryngoscopy performed	66 (43.1%)
VFMI at last follow-up	31 (47.0%)

*PDA* Patent ductus arteriosus, *BTT* Blalock–Thomas–Taussig, *LVAD* Left ventricular assist device, *ECMO* Extracorporeal membrane oxygenation, *VFMI* Vocal fold motion impairment

Of the 322 patients, 105 (32.6%) did not require tube feeding during their recorded clinical course and were excluded as they were not the subject of this research. There were 217 patients that were tube feeding dependent at initial discharge. Of these patients, 110 (34.2%) advanced to a full PO diet, while 107 (33.2%) remained on tube feeds for either primary or supplemental nutrition at the end of follow-up (median 7.2 (IQR 1.5–17.3) months) (Fig. 1).

## Instrumental Swallow Study Evaluation

There were 137 patients that had an instrumental swallow study after initial discharge, and this provided more objective dysphagia information beyond tube feeding status (Table 2). The final instrumental swallow evaluation occurred a median 7.2 (IQR 2.7–16.5) months after the initial dysphagia diagnosis. The median DOSS score was 4 (IQR3-5). As expected, the DOSS score was worse in patients that were still requiring tube feeding at the end of follow up (*p* = 0.0003). The 77 patients that were requiring tube feeding included 58 (75.3%) with tube feeding as the primary diet and 19 (24.7%) patients with tube feeding as the supplemental diet. Thickened liquids were utilized in 27 (45%) patients not on tube feeds and in 47 (61%) patients on tube feeding as a supplemental or primary diet. Only 5 (6.5%) patients were not able to safely tolerate any PO diet at the end of follow-up.

**Table 2:** Final instrumental swallow study data

	All patients	Improved to PO diet	Persistent tube feeding	Univariate *P*-value
Number of patients (N)	137	60	77	
Severity of dysphagia (initial)	Median 4 (IQR 2–5)	Median 3 (IQR2-4)	Median 3 (IQR2-5)	0.6339
Severity of dysphagia (DOSS) on last available swallow study	Median 4 (IQR 3–5)	Median 5 (IQR4-5.5)	Median 4 (IQR3-5)	**0.0003**
Surgery to final swallow (months)	7.2 (IQR 2.7–16.5)	14.2 (IQR2–17.6)	7.2 (IQR3.7–15)	0.8165
Tube feeds primary diet	58 (42.3%)	0 (0%)	58 (75.3%)	
Tube feeds supplemental diet	19 (14.1%)	0 (0%)	19 (24.7%)	
Thickened diet	74 (54.8%)	27 (45%)	47 (61.0%)	
No PO diet	5 (3.7%)	0 (0%)	5 (6.5%)	

Bold value denote statistical significance at the *p* < 0.05

*DOSS* Dysphagia outcome and severity score

## Time to Full PO Diet and Vocal Cord Recovery

**Fig. 3:**
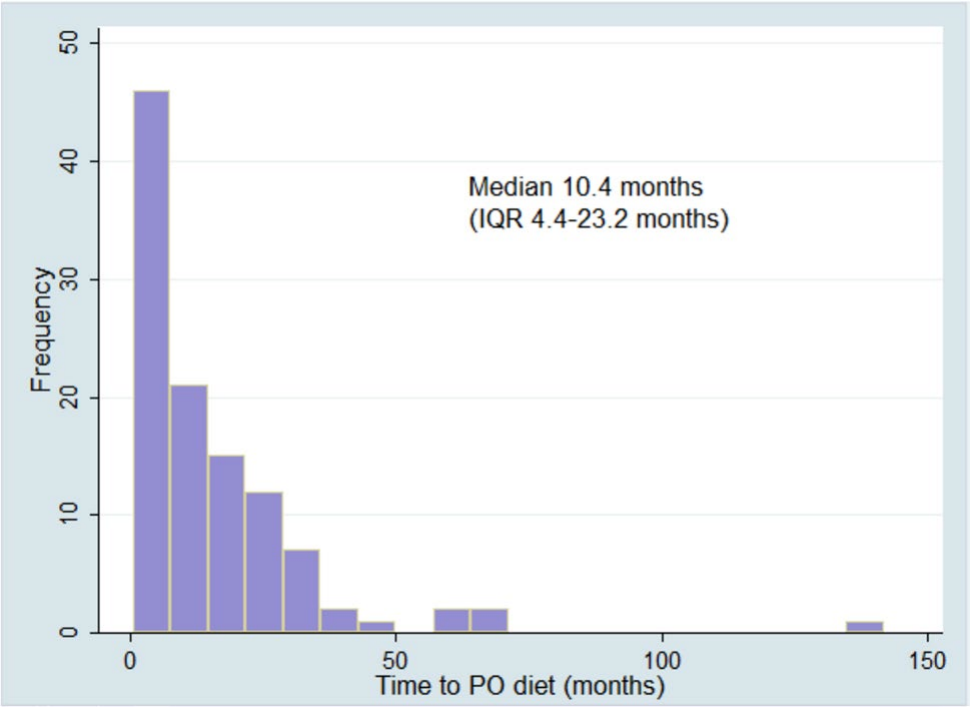
Time to full PO diet in patients that were on tube feeding at initial discharge

**Fig. 4:**
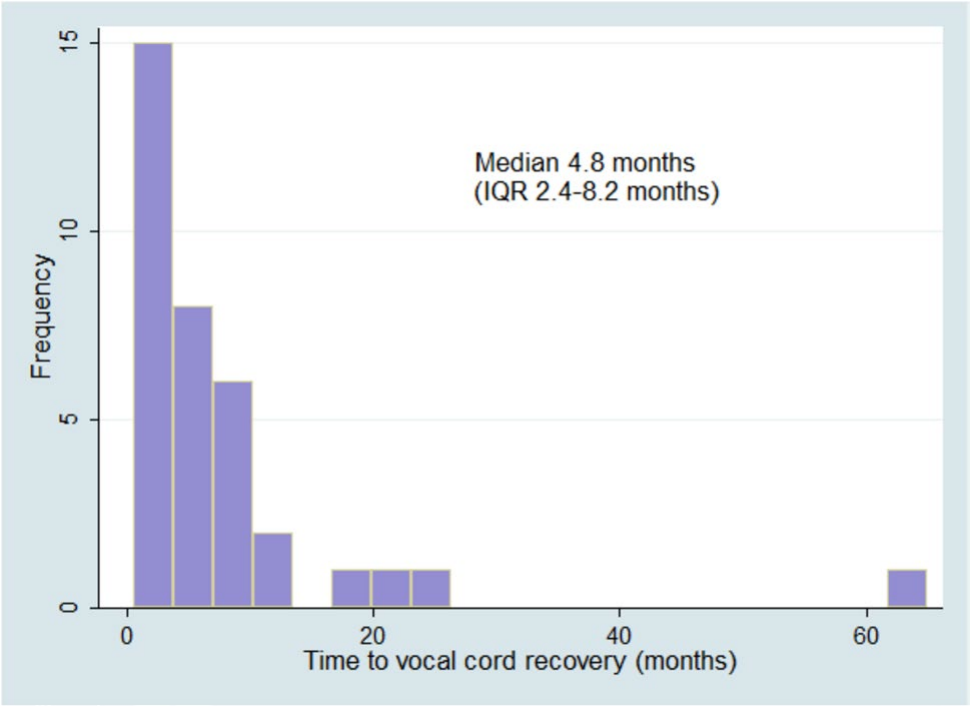
Time to vocal fold motion impairment (VFMI) recovery

There were 110 (34.2%) patients who initially required a primarily tube fed diet were able to achieve a full PO diet at the end of follow-up. The median time to a full PO diet was 10.4 (IQR 4.4–23.2) months after surgery (Fig. 3). VFMI recovery occurred in 35 patients in a median of 4.8 (IQR 2.4–8.2) months after surgery (Fig. 4). Persistent VFMI was not statistically associated with persistent tube feeding at the end of follow-up (Table 3). However, there was a statistically significant correlation between the time it took patients to safely advance to a full PO diet and the time to recovery of VFMI. Patients that took longer to have recovery of VFMI also took longer to be cleared for a safe PO diet (Pearson correlation coefficient 0.77, *p* = 0.0001).

**Table 3:** Factors associated with diet improvement in those with tube feeds at initial discharge

	Improved to PO diet	Persistent tube feeding	*P*-value
N	110	107	
Age at surgery (months)	0.33(IQR0.16–1.7)	1.2(IQR0.23–7.8)	**0.004**
Female Gender	52 (47.3%)	50 (46.7%)	0.936
Prematurity	10 (9.1%)	12 (11.2%)	0.604
Single ventricle cardiac disease	57 (51.8%)	41 (38.3%)	**0.046**
Surgical procedure			
PDA ligation	2 (1.8%)	5 (4.7%)	0.234
Norwood	22 (20%)	16 (15%)	0.328
PDA stent/PA banding	13 (11.8%)	10 (9.4%)	0.554
BTT shunt	5 (4.5%)	16 (15%)	**0.01**
Aortic root	0 (0%)	0 (0%)	
Coarctation of Aorta repair/arch repair	38 (34.5%)	12 (11.2%)	** < 0.0005**
Vascular ring repair	3 (2.7%)	3 (2.8%)	0.973
Arterial switch operation	10 (9.1%)	10 (9.4%)	0.948
Truncus arteriosus repair	3 (2.7%)	2 (1.9%)	0.674
Heart transplant	11 (10%)	13 (12.1%)	0.614
LVAD	2 (1.8%)	17 (15.9%)	** < 0.0005**
Other	1 (0.9%)	3 (2.8%)	0.3
Comorbidities			
ECMO	1 (0.9%)	6 (5.6%)	**0.05**
Syndromic	13 (11.8%)	27 (25.2%)	**0.011**
Neurological	43 (39.1%)	54 (50.5%)	0.092
Airway abnormality	11 (10%)	17 15.9%)	0.196
Lower airway	6 (5.5%)	23 (21.5%)	**0.001**
Tracheostomy	9 (8.2%)	18 (16.8%)	0.054
Nissen	1 (0.9%)	3 (2.8%)	0.3
Complications			
Chylothorax	26 (23.6%)	27 (25.2%)	0.784
Diaphragm paralysis	9 (8.2%)	11 (10.3%)	0.593
VFMI			
VFMI after surgery*	45/70 (40.9%)	31/56 (29.0%)	0.309
VFMI at the end of follow-up*	19/37 (17.3%)	11/25 (10.3%)	0.57

Bold values denote statistical significance at the *p* < 0.05

*PDA* Patent ductus arteriosus, *BTT* Blalock–Thomas–Taussig, *LVAD* Left ventricular assist device, *ECMO* Extracorporeal membrane oxygenation, *VFMI* Vocal fold motion impairment

*Denominator denotes patients that had laryngoscopy performed

## Factors Associated with a Patient’s Safe Return to Full PO Diet

The 110 patients who were able to advance from a primarily tube feeding diet to a safe PO diet were compared to the 107 patients who had a persistent need for tube feeding at the end of follow up. Both univariate analysis (Table 3) and multivariable logistic regression (Table 4) were performed to assess factors that could contribute to persistent need for tube feeding. Several clinical factors including undergoing a BTT shunt procedure (OR 5.0, *p* = 0.005), use of left ventricular assist device (LVAD) (OR6.2, *p* = 0.021), and presence of a genetic syndrome (OR3.6, *p* = 0.003) were associated with the persistent need for tube feeding. Older median age at surgery at the time of surgery, ECMO use, lower airway abnormality, clinical signs of aspiration on the preoperative swallow assessment, and a delay in obtaining the first swallow study after surgery were associated with persistent tube feeding only on univariate analysis. Interestingly both single ventricle cardiac disease (OR0.38, *p* = 0.005) and coarctation of the aorta/aortic arch repair (OR 0.20, *p* < 0.0005) were more predictive of improvement to a PO diet at the end of follow-up.

**Table 4:** Multivariable logistic regression models for factors associated with persistent tube feeds at end of follow-up

Variables	Odds ratio (95% confidence interval)	*P*-value	c-statistic/area under ROC
Single ventricle cardiac disease	OR 0.38 (0.20–0.75)	***p* = 0.005**	0.7704
Blalock–Thomas–Taussig Shunt	OR 5.0 (1.6–15.2)	***p* = 0.005**	
Coarctation of Aorta repair/arch repair	OR 0.20 (0.085–0.45)	***p* < 0.0005**	
LVAD (left ventricular assist device)	OR 6.2 (1.3–29.2)	***p* = 0.021**	
Genetic Syndrome	OR 3.6 (1.5–8.2)	***p* = 0.003**	

Bold values denote statistical significance at the *p* < 0.05

Multivariable logistic regression models associated with feeding tube need. A stepwise procedure was used for multivariable model construction, with a *P*-value of < 0.15 (on univariable analysis) to enter the model and a *P*-value of 0.05 to remain in the model

## Discussion

This study evaluated 322 mostly infant patients who underwent surgical repair of CHD to better understand their dysphagia, its time course, and its relationship to VFMI. Severe dysphagia is common in this patient population but there was a significant improvement over time. At initial discharge, 110 (34.2%) patients who were on tube feeding demonstrated substantial improvement in their dysphagia over time and were ultimately able to advance to a full PO diet in a median 10.4 months (IQR 4.4–23.3). However, 107 (33.2%) patients continued to have severe dysphagia requiring tube feeding at the end of study follow-up (median 7.2 (IQR 1.5–17.3) months).

We found that VFMI persistence was not associated with the need for tube feeds; VFMI recovery typically occurred a median of 4.8 months (IQR2.4–8.2) after cardiac surgery which was several months sooner than return to a full PO diet. This suggests that there are additional factors beyond VFMI recovery that impact the persistence of dysphagia and transition to a PO diet in children with CHD.

The prevalence of VFMI and dysphagia with aspiration in children with CHD after cardiac surgery has been assessed in multiple studies with VFMI rates from 1.7 to 65% and 45 to 64% of these patients were also noted to have aspiration [11, 13, 14, 18]. The reason for this variance is unclear and may depend on the disease process itself or the timing and frequency of the assessment of the vocal cords. There is no universally accepted protocol for vocal cord assessment after cardiac surgery. Some protocols assess based on clinical symptoms and others utilize timed universal screening. At our institution we performed laryngoscopy based on either clinical symptoms (voice changes) or if aspiration was noted on an instrumental swallow evaluation. Limited data are available on the time course of recovery of VFMI. In one study 68.8% of their patients had complete recovery in 106 days while in another 28% recovered in 4.3 months [19, 20]. Recovery of VFMI may take up to two years after the suspected injury date [21]. There is even less data available on the resolution of dysphagia in children with CHD after cardiac surgery. Recovery rates range from 6.6 weeks in 50% of patients in one study, 230 days in another, and 1.3 ± 1.67 years in yet another study [13, 14, 18]. This wide variation is due to multiple factors including the complexity of this patient population and the quality of the studies with retrospective analysis with small patient groups/cohorts predominating. In addition, many studies did not use clinical or instrumental swallow evaluations to assess the time course to recovery.

Our study did not find an association between VFMI and persistent need for tube feeding. Similarly, a 2016 study reviewed unilateral VFMI that resulted from several etiologies, including cardiothoracic surgery, idiopathic VF dysfunction, prolonged intubation, central nervous system disorders, and other iatrogenic injury to the recurrent laryngeal nerve [12]. The authors found that 42.6% of patients had recovery of VFMI and saw an increase from 31.5% to 79.5% of patients who were fed orally at the time of follow-up, suggesting that a direct correlation between recovery of VFMI and recovery of swallow function was not demonstrated. However in their study, the dysphagia definition was different and patients fed both orally and with tube feeding were included in the tube feeding group [12]. Looking further, we did find a statistically significant correlation between time to full PO diet and time to vocal cord recovery. Patients that took longer to demonstrate VFMI recovery also took longer to be cleared for a PO diet. This helps provide prognostic information regarding dysphagia recovery and helps set expectations as often caregivers are frustrated that dysphagia persists despite VFMI recovery.

Understanding the severity of dysphagia and VFMI status may additionally help providers make a safe PO feeding plan for discharge home. This is particularly important for those patients with single ventricle physiology that will be discharged home during interstage monitoring. These patients had a high dysphagia burden as indicated by the DOSS score, both on the initial VFSS DOSS score (median 4, IQR 2–5), and the final DOSS score (median 4, IQR3-5). There was a trend toward a higher rate of initial VFMI in the single ventricle population (OR 1.8 CI 0.94–3.5 *p* = 0.054). However, there was no statistical association between single ventricle disease and vocal cord recovery. In addition, the time to a PO diet took longer in the single ventricle patients (median 15.8, IQR 8.6 to 23.8) compared to the biventricular patients (median 5.5, IQR 2.4 to 12.6, *p* < 0.00005). Single ventricle patients are considered to be at higher risk for developing necrotizing enterocolitis given reduced systemic blood flow [6]. Thickened liquids were utilized as a compensatory strategy in 45% of our patients that did not require tube feedings and in 61% of patients that were fed by mouth with supplemental nutrition via tube. Seventy-six percent of the single ventricle patients were on thickened liquids at the end of follow-up. More research is needed to help guide the use of thickened liquids for the single ventricle population including the variation in practice and the use of various thickening agents in the setting of an increased risk for bloody stool, milk protein intolerance, and necrotizing enterocolitis. Having the ability to recommend thickened liquids with close medical follow-up may potentially provide more opportunities for patients to be either partially or fully orally fed. Additionally, having knowledge of when VFMI may recover can help to guide timing and frequency of repeat instrumental evaluation in the out-patient setting. Knowing that there can be as much as a 6-month lag between VFMI recovery and transitioning to full oral PO feedings can help medical providers utilize resources to conserve both medical costs for caregivers and radiation exposure of patients.

Discharge with nasogastric tube feeding for either primary or supplemental nutrition can place emotional and financial stress on parents, impact options for childcare/return to work, increase risk for hospital readmission and influence the parent child relationship [22–25]. The results from this study suggest that patients with a genetic syndrome, LVAD or BTT shunt surgical history have a higher association with long-term tube feeding needs;. When parents and patients are receiving counseling to prepare for cardiovascular surgery, it is important to provide information about these factors so that it may help set expectations and allow caregivers to make decisions about long-term alternative feeding methods.

The multivariable model also demonstrated that coarctation/arch repair procedures were less likely to be associated with tube feeding at the end of follow-up. This is an interesting finding as previous studies have noted the opposite although they have not assessed long-term dysphagia outcomes [10, 26]. We postulate that this patient group has a better long-term dysphagia outcome because the cardiac defect will have little residual hemodynamic concern once repaired. By contrast those with more complex disease such as those with a genetic syndrome, BTT shunt or LVAD placement have more complex and protracted hospital stays, comorbidities and residual hemodynamic lesions that impact feeding tolerance. In addition, a cardiac defect that is fully surgically corrected makes the patient a better candidate for compensatory feeding strategies such as use of thickened liquids (i.e. lower risk of NEC) which will allow them to transition to full oral feeding. Furthermore, on multivariable analysis it was demonstrated that patients with single ventricle cardiac disease had a lower association with tube feeding at the end of follow-up (OR 0.38 (0.20–0.75, *p* < 0.005) even though on initial post-cardiac surgery discharge single ventricle patients are more likely to go home with tube feeds. This is likely related to a standardized tube weaning program that is included in the single ventricle care pathway after recovery from a Glenn procedure or Bi-ventricular repair. Wong and colleagues describe data collected from six centers that used a standardized hunger provocation tube weaning process for 64 single ventricle patients after the interstage period. Of the 64 patients, 60 (94%) patients were successfully weaned from tube feedings within a median time of 12.5 days [27]. This included patients with documented aspiration, diagnosis of genetic syndrome, and extracardiac diagnoses. This data may help to drive cardiovascular surgery programs to incorporate tube weaning protocols into post- operative care pathways to help patients transition off tube feedings, regardless of the type of cardiac lesion or additional comorbidities.

## Limitations

This retrospective study includes limitations related to its descriptive nature and lack of a comparative control group of patients. Although representative of the varying diagnoses, patient age, types of surgical intervention, and complications encountered in a large acute pediatric cardiac unit, the results of this study may be difficult to generalize given the variety of cardiac procedures that were completed. While we included patients aged 0–21 in our study inclusion criteria, the data are more representative of younger patients due to automatic SLP orders for surgical patients < 12 months of age and infants are more likely to need dysphagia management with tube feeds compared to older patients. In addition, data may be limited in generalizability as not all patients underwent laryngoscopy to identify VFMI or instrumental swallow study after surgical repair and the decision to perform these evaluations was instead based on symptoms. Given the multifactorial nature of dysphagia in the cardiac patient population, this study does not address other potential factors that may impact dysphagia recovery including preoperative feeding status, initial hospital length of stay, intubation time, complicated postoperative course, patient maturation and acquisition of feeding skills, improvements in motor development, changes in respiratory stability, or compensation of the contralateral vocal fold for improved airway protection.

## Conclusion

Patients that require surgical intervention for CHD are at risk for both postoperative VFMI and dysphagia. VFMI recovery is not associated with dysphagia recovery and dysphagia tends to persist for several months after VFMI resolves. Understanding this difference in time course to recovery and the factors identified that are associated with long-term need for tube feeding will help guide provider recommendations and counseling for caregivers to set expectations regarding feeding plans.

## Data Availability

No datasets were generated or analysed during the current study.
